# Experiential Education in Pharmacy Curriculum: The Lebanese International University Model

**DOI:** 10.3390/pharmacy9010005

**Published:** 2020-12-29

**Authors:** Marwan El Akel, Mohamad Rahal, Mariam Dabbous, Nisreen Mourad, Ahmad Dimassi, Fouad Sakr

**Affiliations:** 1 Pharmacy Practice Department, Lebanese International University, Beirut 961, Lebanon; mariam.dabbous@liu.edu.lb (M.D.); nisreen.mourad@liu.edu.lb (N.M.); ahmad.dimassi@liu.edu.lb (A.D.); 2 School of Pharmacy, Lebanese International University, Beirut 961, Lebanon; mohamad.rahal@liu.edu.lb; 3 PharmD Program, Lebanese International University, Beirut 961, Lebanon; fouad.sakr@liu.edu.lb

**Keywords:** pharmacy practice, experiential education, learning consistency, Lebanon

## Abstract

Experiential education is an essential component of pharmacy education in order to allow intern students to experience real conditions and training opportunities in different inpatient and outpatient settings. This paper provides a description of the pharmacy practice experiences (PPEs) in the Lebanese International University (LIU) 5-year bachelor of pharmacy (BPharm) and postgraduate doctor of pharmacy (PharmD) programs; focuses on the opportunities and challenges encountered; and presents a model for experiential education in Lebanon. Learning outcomes and thus students’ acquisition of predefined competencies are evaluated in actual practice settings through assessment tools. Our experiential education program aligns with the accreditation/certification criteria set by the Accreditation Council for Pharmacy Education (ACPE) and equips future pharmacists with the knowledge and skills to become major components in the healthcare team.

## 1. Introduction

Pharmacy education globally aims to prepare competent patient-centered pharmacists who are able to optimize patient care and provide medication therapy services in different settings [1,2]. Experiential learning is a fundamental element in pharmacy education [3]. It relies heavily on practice-based experiences to provide direct contact with patients and training opportunities in real conditions [4]. However, meeting considerable experiential curricular necessities can be challenging and demanding with expanded students’ enrollments and changes in the healthcare system [5].

Schools of pharmacy are now required to integrate experiences into their curricula to develop the expected practice competencies [1]. For this reason, practice experiences in the professional years of the curriculum permit students, under the supervision of a preceptor, to experience the direct responsibilities of patient care, and to lead into advanced experiences. Consequently, advanced experiences in the postgraduate doctor of pharmacy (PharmD) program, which is an additional one year in Lebanon after the BS degree under the supervision of practicing pharmacist and faculty preceptor, allow students to get involved more in case discussion, decision-making, and in direct patient care [6]. These experiences take place in different inpatient and outpatient settings to broaden the practice exposure of students and to achieve all desired outcomes of experiential learning [6].

Nevertheless, schools of pharmacy are frequently challenged to find practice placements for their students [7]. The schools of pharmacy in the United States have encountered a number of challenges to identify, develop, and sustain practice experiences to meet the learning outcomes of experiential education [8]. Challenges are confronted in recognizing competent practice sites, finding sites in geographical regions around the students’ areas of residency, and providing the necessary training and development to preceptors [9,10,11].

In Lebanon, there are five schools of pharmacy that follow a diversity of models between the American, Canadian, and French systems [12]. Experiential education is a key element in these different systems and curricula, yet the five schools provide experiential learning at different qualities and quantities. According to the Lebanese pharmacy curricular philosophy, experiential education is a must to acquire the standard competencies by a pharmacist [13]. It involves considerable time commitment from preceptors to teach, mentor, and evaluate students during the different experiences, and to guide the application of didactic knowledge into real practice [14,15].

The School of Pharmacy at the Lebanese International University (LIU) was established in 2002, with a mission to educate and train students to become distinguished professionals in pharmacy practice, research, and community services. Several difficulties were confronted by the school, such as finding competent practice sites near students’ regions of residency, training, and developing preceptors to provide excellent supervision during clinical practice. To overcome these challenges, the school afforded new units and hospitals for case diversity and provided continuous workshops, lectures, and training about professional education for the onsite preceptor. Similarly, the school offers faculty development workshops, preceptor development, updates in pharmacy practice, and program formation and implementation for the faculty preceptors. Therefore, after two decades, the BPharm program is fully compliant with the Accreditation Council for Pharmacy Education (ACPE) standards and quality criteria, and it is currently the only ACPE certified program in Lebanon as of August 2020. In fact, the school adopts the American model to offer a 5-year bachelor of pharmacy (BPharm) program and later on the postgraduate doctor of pharmacy (PharmD) program. As such, the BPharm program allocates a minimum of 300 h for Introductory Pharmacy Practice Experiences (IPPEs) in the first two years of the program and a minimum of 1140 curricular hours for Pharmacy Practice Experiences in the three professional years, resulting in a total of 1440 h. The PharmD program is a full-time practice program that provides Advanced Pharmacy Practice Experiences (APPEs) in clinical settings mainly and with interprofessional learning and experience [6].

There is a lack of published literature that reflects the reality of pharmacy experiential education in Lebanon. Thus, this paper reports a snapshot of the pharmacy practice experiences in the LIU BPharm and PharmD programs, the opportunities, encountered challenges, and provides a model for experiential education in Lebanon.

## 2. Community Setting

Introduction to practice in the community setting starts early in the first two years of the program and continues in the first and the second professional years (third and fourth years of the program) through a series of twelve-week practice experiences in each year known as Pharmacy Practice Experiences I and II (PPE I and PPE II). These courses introduce students to the philosophy of pharmaceutical care practice including patient counseling, monitoring plans, and patient outcomes, with emphasis on the role of the pharmacist as the primary manager of patient drug therapies. The community pharmacy rotations allow students to apply the foundational knowledge acquired through didactic and simulated courses in real practice settings, and exhibit interprofessional skills to communicate efficiently with members of the healthcare team. The rotations also allow students to demonstrate behaviors and values that are consistent with the trust given to the profession by patients, other healthcare providers, and society, as well as to demonstrate attitudes that are respectful of different cultures, and to operate as a pharmacist in a real practical setting and identify the role of the pharmacist [16].

Each PPE (PPE I and II) course has a structured manual to guide students and preceptors to achieve the intended learning outcomes. The manuals are divided into different modules that allow students to acquire the essential knowledge, skills, attitudes, and values to be effective members through an educational experience augmented by collaboratively working with actual or simulated healthcare professionals and practitioners.

PPE I modules aim to provide students with an introduction to basic pharmacy practice skills, to understand the fundamental healthcare needs of diverse populations, and to foster a sense of professional responsibility to help meet those needs and provide services. These include activities that incorporate patient-encounter exercises, including training on procedures such as blood pressure, glucose, and cholesterol measurements. On the other hand, the PPE II course expands into more advanced topics related to patient care in community settings. In this course, students apply their knowledge and skills in topics related to noncommunicable and communicable diseases. Collectively, these learning experiences provide students with adequate basic and advanced community knowledge and communication skills to engage in patient care for diverse populations and interact with healthcare professionals.

Ryan TJ et al. described the fourth- and fifth-year experiential learning placement where in each year students are required to undertake three online modules. In the fourth year, they target three of the six Core Competency Framework for Pharmacists (CCF) domains including personal skills, professional practice, organization, and management skills, while in the fifth year they target all six CCF domains including the remaining domains of practice: safe supply of medicines; safe and rational use of medicines; public health [16].

## 3. Inpatient Setting

Inpatient clinical rotations are introduced in the BPharm program during the third professional year (fifth year of the program) in two simultaneous practice courses known as Pharmacy Practice Experiences III and IV (PPE III and IV). These courses are a series of fifteen-week rotations within a tertiary hospital, offered during the fall, spring or summer semesters; they are designed to provide the opportunity to the students to further develop their clinical skills in pharmacy practice.

In fact, these clinical PPE courses are intended to provide senior pharmacy students with an adequate practice exposure to apply their academic knowledge, problem solving, and decision-making skills [17]. Thus, this will enable the pharmacy interns to provide patient care, drug dispensing, drug information, health promotion/disease prevention, and other pharmacy services [18]. Similarly, these courses aim to develop the pharmacy students’ skills to practice as part of the healthcare team, and interact with patients and other healthcare professionals to identify, resolve, and prevent drug therapy related problems, therefore ensuring safe, effective, and economical drug therapy [19].

In our model, each student completes one “Hospital Pharmacy Rotation”, three major clinical rotations, and one elective rotation. The length of each rotation is three weeks. During the three-week period of hospital pharmacy rotation, each student will be under the direct supervision of the onsite hospital/clinical pharmacist and the assigned faculty member preceptor. Additionally, each student shall fill the hospital pharmacy manual that contains seven objectives and a total of 12 order screenings [20]. For instance, the students prepare medications precisely and safely to the in-patients, according to the hospital’s guidelines. Additionally, they illustrate a drug utilization review and patient counseling, and recognize and describe the job of the hospital pharmacist and pharmacy technician in each component of medication use system in different practice settings [21]. Furthermore, the students identify potentially significant drug–drug, drug–food, drug–laboratory, and drug–disease interactions, recommend drug discontinuation or dosage alteration when indicated, provide pharmacokinetic consultation for agents requiring such monitoring, provide educational presentations to pharmacy staff, students, and other healthcare professionals, participate in research activities, demonstrate initiatives in the hospital pharmacy setting, such as checking and updating formularies, monitoring, and patient education [22,23].

The remaining twelve weeks are referred to as the “Clinical Care Rotations”, during which the students are assigned to a medical team in different wards [24]. The students actively participate in daily rounds with the medical team in different units including Cardiac Care Units (CCUs), Intensive Care Unit (ICU), and Internal Medicine and Pediatrics to complete their major rotations. Selection of the elective rotations is tailored according to availability of the services and vacancies, and could include Oncology, Endocrinology, Infectious Diseases, Psychiatry, Nephrology, Dermatology, and Geriatric departments. DeAngelis and Wolcott described a preceptor model, in which three critical tasks were provided to the pharmacy interns by the preceptors in the training setting. These tasks are related to professionalism in stressful situations, commitment to service, and ethical and moral decision-making [25].

## 4. Outpatient Department (OPD)

The OPD rotations aim to provide the students with the necessary skills to practice in ambulatory care. This setting has a set of services provided to ambulatory patients, home care patients, hospital staff, and probably emergency department patients depending on the level of care of an organization [26]. In other words, the terms outpatient or ambulatory refers to patients not occupying beds in hospitals or other inpatient settings. This rotation prepares future pharmacists to play an essential role in the safe, quality, and effective use of medications in improving patient’s physical and mental wellbeing [27].

## 5. The Doctor of Pharmacy (PharmD) Program

The PharmD program is a clinical-based program that is delivered over one academic year over two semesters. Each semester includes community and both major and elective clinical rotations based on each student’s specified schedule. In concert with the core values of the School of Pharmacy at LIU and in-line with ACPE standards, the program’s philosophy is to provide the students with a comprehensive foundation in advanced pharmacy practice experience (APPE) and advanced clinical pharmacokinetics, as well as the necessary research skills in drug utilization, optimization, and safety [28].

The APPEs in the program consist of six clinical rotations and one community rotation. The advanced practice clinical rotations are further divided into major and elective rotations. The length of each rotation is one month, and students will complete four required major rotations and two selected elective rotations, as well as one community rotation. The major APPE rotations include the cardiac care unit (CCU), intensive care unit (ICU), internal medicine (IM), and pediatrics (Ped). The elective rotations will focus mainly on infectious diseases (IDs), hematology and oncology, nephrology, geriatrics, and psychiatry as well as emergency settings (EDs). These APPE rotations should reinforce the development of the skills and knowledge students received during their BPharm curriculum [29]. The APPEs also provide students with the opportunity to serve patient populations in a variety of settings and to collaborate with other healthcare professionals. These experiences offer exposure to patients and disease states that clinical pharmacists are likely to encounter in practice [30]. The students will work under the supervision of a licensed doctor of pharmacy, the preceptor, at all times during the APPEs.

The goals and objectives for each experience are based on the competencies needed to fulfill the requirements of the PharmD program [31]. Students must demonstrate an advanced level of proficiency in each competency by the end of the experiential rotation in order to successfully complete the rotation [32]. Moreover, the students will be actively involved with the medical team in the clinical rounds on the hospital floors, based on each major or elective rotation, to achieve the goals of the program [33].

## 6. Preceptors

The LIU model of experiential education includes a dual-preceptor concept, in which each student will be under the close supervision of the onsite preceptor and the faculty preceptor. Precepting is a foundation of experiential education by promoting the competencies needed by student pharmacists to succeed in a new environment [34]. The concept of precepting has been adopted by the LIU, so in addition to the hired academic staff who mainly possess precepting duties, the school of pharmacy is affiliated with a number of adjunct (onsite) preceptors in community and hospital settings across the Lebanese districts to fulfill the experiential education needs of the students. The role of the faculty preceptors is to continuously communicate with the onsite preceptors regarding students’ performances, to address their concerns, and to assist them to optimize the students’ clinical learning environment. In addition, faculty preceptors act as connectors between onsite preceptors and the school of pharmacy. With respect to onsite preceptors, LIU provides them with a comprehensive development program, which includes live seminars that provide continuing education credits, webinars, triannual newsletters, and onsite visits, which all aim to provide continuous training [35,36,37]. The site preceptors, in turn, develop and sustain model experiential learning opportunities for their students [38]. Together, faculty and onsite preceptors facilitate the most favorable learning environment for the students to meet personal and course objectives. They direct students to resources and evidence-based readings and answer their inquiries. They also act as socializers to foster the development of cultural competency among students, and, more importantly, as role models who possess leadership and professional skills.

## 7. Responsibilities and Duties

Each student is responsible for preparing two active cases per week to be thoroughly assessed following the SOAP (Subjective, Objective, Assessment, Plan) format, and to monitor five to seven active patients and at least two documented interventions per week. The students discuss their collected cases with their preceptor, who assesses their performance according to the case discussion rubric [39]. For instance, the rubric includes points about completion of several tasks such as general interpretation of the case, collection, identification, and interpretation of the necessary subjective and objective data [40]. In the assessment part, the rubric includes points about the definition of the disease and its risk factors, identification and assessment of each problem, evaluation of the suitability of drug therapy, selection of the most updated and relevant resources, application of the best evidence to individualize patient therapy, inclusion of the desired therapeutic goals, and recommendations for drug and nondrug therapy [41]. Finally, in the plan part, the rubric includes points about recommendations for each problem, monitoring, follow-up plan, and counseling tips for all the medical problems [42,43].

Moreover, each student presents one recent article during the rotation. The article is evaluated and presented by the student to the preceptor in the presence of the other students to ensure a group discussion. Each student additionally presents one topic in a PowerPoint presentation during the rotation in the presence of the other students to exchange thoughts and ideas [44]. Likewise, students are required to attend and participate in grand rounds, meetings, talks, and seminars conducted in their services, especially those pertinent to pharmacy practice.

## 8. Competency-Based Assessment

PPEs are structured and delivered to allow the students to acquire the competencies of different domains of the program’s learning outcomes. The reflected domains include domain 1: foundational knowledge, assessment of medicines, compounding of medicines, and dispensing; domain 2: pharmaceutical care, patient-centered care, medication use systems management, and promoter and care provider; domain 3: essentials for practice and care, problem solver, educator, advocate, collaborator, scholar, culture sensitivity, and communicator; domain 4: approach to practice and care, practices professionally, practices legally, practices ethically; domain 5: professionalism, honesty and liability, adherence to ethical principles and moral reasoning in relation to patients, other healthcare professionals and society; domain 6: personal and professional development, which fosters self-awareness, leadership and management skills, and innovation.

Assessment tools are utilized in actual practice settings during PPEs to evaluate students’ acquisition of these competencies. A student’s ability to perform patient-centered care skills and professional attitude is assessed by assigned faculty member preceptors through onsite discussions and grading that relies on performance-based rubrics. The value of the rubric is in both the standardization of criteria for grading and in the definition of an appropriate performance for students [45]. Students are provided with feedback of their performance after each graded activity which allows the student to self-assess and reflect on a particular task [46]. Faculty preceptors offer a balance of positive observations and recommendations for improvement. They provide constructive formative and summative feedback on students’ performances to improve them. In fact, preceptors are the cornerstones in helping students achieve their experiential experience goals through guiding and coaching them to develop the knowledge, clinical skills, and professional attitudes to succeed in their community rotations [47].

The ability of students to utilize available technology to optimize the efficacy and safety of the medication use systems, to demonstrate time management and interprofessional communication skills, is evaluated by onsite preceptors. Moreover, the faculty member preceptors conduct workshops to evaluate student group sessions regarding selection of appropriate medication use and care for studied situational cases. Those workshops allow group discussion, sharing experiences, and cooperative learning. At the end of PPE I and II experiences, a comprehensive case-based final written exam is conducted to evaluate students’ overall knowledge skills and abilities. This strategy allows us to assess competencies in a planned and structured way [48]. On the other hand, and at the end of the PPE III and IV courses, each student will reflect on his/her culminating practical experiences by writing an essay and filling a site evaluation rubric, as well as his/her ability to assess self-awareness and personal development [49,50]. Examples of students’ reflective writings are presented in Table 1.

## 9. Practice Sites

The Pharmacy Practice Department has established an adequate number and mix of qualified practice sites to support the curricular pharmacy practice experience. All affiliated community pharmacies are accredited by the Lebanese Ministry of Public Health (MoPH) and have been established for at least 3 years; additionally, all community pharmacists fulfilled their Continuing Education (CE) credits as set by the Lebanese Order of Pharmacists (OPL). The department always ensures that the affiliated community pharmacies have a patient-centered mission and foster an appropriate environment to deliver quality services. Within the pharmacy, they use information systems and technology to support quality service delivery, drug utilization, and safe medication distribution. Moreover, the pharmacist should be available to help students achieve the goals during their internships along with sufficient professional, technical and support staff resources to fulfill the mission of the practice.

Experiential education sites were evaluated periodically through site evaluation forms that were filled by the students to assure that the essential components of good practice sites were still met and/or improved. Table 2 provides information on the community pharmacy sites from the site evaluation survey from the year 2017 through to 2019. The majority of students either strongly agreed or agreed with the quality of practice sites; the highest of these responses was for the statement “the site provided a practice environment that nurtures and supports pharmacist and student interactions with patients”, which ensured that the students are practicing in highly qualified practice sites. The lowest percentage of responses for the question “the site allowed me to participate in billing third parties for pharmacy services”; this is because in Lebanon billing third parties as a pharmacy service is not available in all practice sites all over Lebanon.

**Figure 1:**
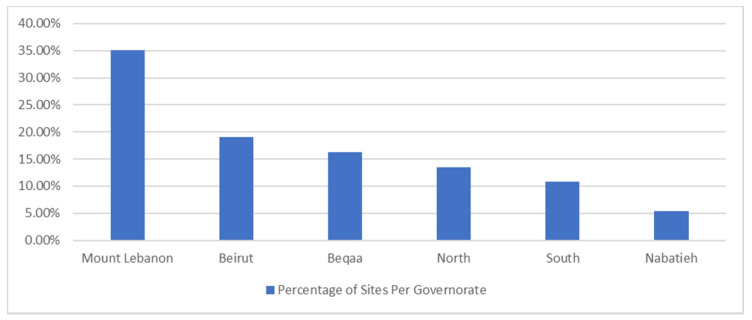
Percentage of Sites Per Governorate.

**Figure 2:**
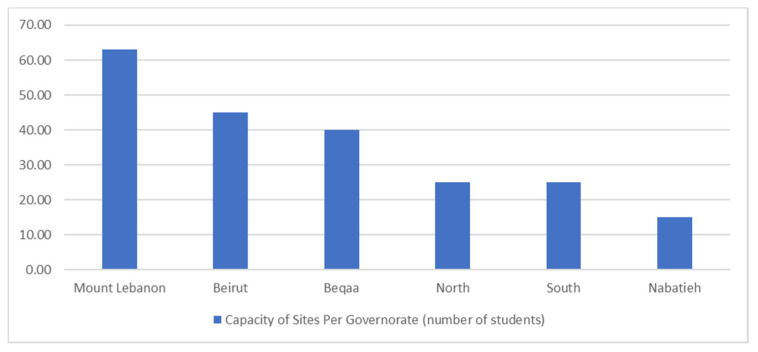
Capacity of Sites Per Governorate (number of students).

Inpatient practices are offered at all governorates of Lebanon. A total of 37 training sites were identified for PPE III and IV internships. Most of the offered sites are found in Mount Lebanon region with 13 hospitals (35.1%), followed by Beirut with 7 hospitals (19%), then Beqaa with 6 hospitals (16.2%), then North area with 5 hospitals (13.5%), then South area with 4 hospitals (10.8%), to end up with Nabatieh with 2 hospitals (5.4%). With respect to site capacity, a total of 213 students can be distributed allover Lebanon. The Mount Lebanon region has the highest capacity with 63 placements (29.5%), followed by Beirut with 45 placements (21.1%), then Beqaa with 40 placements (18.7%), then North and South areas equally with 25 placements each (11.7%), and Nabatieh with 15 placements (7.3%). The percentage of practice sites per governorate and capacity of sites are demonstrated in Figure 1 and Figure 2, respectively.

## 10. Strengths and Limitations

The experiential learning comprises placements in a variety of practice settings in Lebanon. The LIU school of pharmacy has established agreements with community, hospital, and ambulatory care practice sites to allow for students’ placements allover Lebanon. The affiliated sites meet the required criteria and allow students to achieve the desired learning outcomes of the practice experiences. A global limitation of experiential education in Lebanon is that the concept of an onsite preceptor is still new, and the pharmacy practitioners still need to further develop their precepting skills. Yet, the dual-preceptorship concept that LIU follows allow to overcome this limitation by depending heavily on the faculty preceptor to fill-in this gap. Another concern arises around that not all practice sites are equivalent in capacity and quality. For this, placements are always weighed and distributed according to the availability of rotations at each site.

## 11. Conclusions

Experiential education is an integral component of the pharmacy curriculum at LIU. The reported style of practice experiences aims to prepare competent and patient-centered pharmacists who are able to practice in inpatient and outpatient settings. It also prepares them to become lifelong learners and expertise in dealing with evolving challenges in providing patient care. In addition, these experiences align with the accreditation/certification criteria set by the ACPE, and meet the expectations of stakeholders that would affect future pharmacist employment. The foundations of this model are also adopted by other schools of pharmacy in Lebanon with modifications specific to the universities. Moreover, it is also followed by other healthcare faculties, such as nursing, dietetics, and physiotherapy, as it offers a solution to healthcare schools that are building up their clinical experience. However, a number of challenges are still encountered to optimize the practice experience. Those challenges stem from the site capacity and quality, especially in rural areas.

## Figures and Tables

**Table 1:** Students’ reflective quotes about their pharmacy practice experience (PPEIII/PPEIV) in the hospital.

“It was a good experience for me, I knew how to apply what I had learned in the past 5 years, and how to discuss the details of patients’ files and how to act whenever there is a change or abnormality in their lab values”
“It was the first time I discuss my information with a specialized doctor especially in the oncology rotation and internal medicine”
“Finally the most important thing is that we learned how to talk with the patients and how to listen to their complaint in a way to help them by decreasing their pain and suffering”
“In these three months I knew how to discuss a medical issue with my friends in the hospital, we discussed our cases together”
“For the first few days, everything was new to us and we weren’t feeling confident about what to do, however, after the first week has passed we started to get along and we began to participate with doctor’s rotations on the floor”
“My rotation at the pediatrics floor was one of the most interesting since I saw many rare cases and gained more information about them, one of them was Joubert Syndrome”
“These 3 months added a lot of experience to our journey and made us more ready to start our next step as official pharmacists”
“In addition to learning and doing cases, we made new friends from different universities and we enjoyed our time and had some fun during our breaks, and on May 9 we participated in an Awareness campaign about cardiac diseases where we prepared 4 posters about Hypertension, Diabetes, Weight loss, and Smoking cessation which was a new experience and we were able to be into more contact with the patients”
“At the pharmacy site I learned many new drugs that are given intravenous such as midazolam, and also how to pack medication for each patient accurately by putting stamps labeled as caution, look alike and sound alike”
“During my rotation in ICU/CCU floor, I had attended a PCI and angioplasty. I have learned more information about many equipment like ventilators and defibrillators.”
“The material there was not very advanced and was not like other hospitals concerning diagnostics, surgery, ER rooms. Even though these were not very advanced, I would say I earned a lot of experience whilst doing training at this hospital”
“The work environment was not the most welcoming to pharmacy interns, unlike other specializations. Also, we were left to figure out a lot on our own, the pharmacy staff was not the guidance that we expected. More so, the role of clinical pharmacist was not fully valued or understood, drug knowledge or expertise were not used or asked for”
“But the disadvantage that there is no space for the students in each floor, mainly in the IM floor.”
“My first rotation was in the oncology department. It was a bad start according to me because cancer means a lot so I wasn’t comfortable while dealing with the cases.”

**Table 2:** Site evaluation survey.

Year of Survey (Percentage of “Strongly Agree” and “Agree” Responses)			
	2017	2018	2019
The site had a patient population that exhibited diversity in culture, medical conditions, gender, and age, where appropriate	81.2	81.9	81.7
The site provided a practice environment that nurtures and supports pharmacist and student interactions with patients	85.5	85.7	85.9
The site was adequately equipped with the technology needed to support student training and to reflect contemporary practice	85.1	85.3	85.7
The site displayed a professional image	85.1	85.1	85.2
The site allowed me to allowed to participated in Processing and dispensing new/refill medication orders	78.9	82.1	81.1
The site allowed me to participate in Conducting patient interviews to obtain patient information	63.1	68.3	72.9
The site allowed me to participate in Triaging and assessing the need for treatment or referral	62.2	65.4	69.4
The site allowed me to participate in Identifying patient-specific factors that affect health, pharmacotherapy, and/or disease state management	68	70.9	70.9
The site allowed me to participate in Assessing patient health literacy and compliance	70.1	72.3	72
The site allowed me to participate in Interacting with pharmacy technicians in the delivery of pharmacy services	77.3	78.2	79.3
The site allowed me to participate in Billing third parties for pharmacy services	45.5	47.8	50.1
